# Effectiveness of a community-integrated intermediary care (CIIC) service model to enhance family-based long-term care for Thai older adults in Chiang Mai, Thailand: a cluster-randomized controlled trial TCTR20190412004

**DOI:** 10.1186/s12961-022-00911-5

**Published:** 2022-11-29

**Authors:** Myo Nyein Aung, Saiyud Moolphate, Thin Nyein Nyein Aung, Yuka Koyanagi, Akrapon Kurusattra, Sutatip Chantaraksa, Siripen Supakankunti, Motoyuki Yuasa

**Affiliations:** 1 Department of Global Health Research, Juntendo University Graduate School of Medicine, Tokyo, 113-8421 Japan; 2 Advanced Research Institute for Health Sciences, Juntendo University, Tokyo, 113-8421 Japan; 3 Faculty of International Liberal Arts, Juntendo University, Tokyo, 113-8421 Japan; 4 Department of Public Health, Faculty of Science and Technology, Chiang Mai Rajabhat University, Chiang Mai, 50300 Thailand; 5 Department of Family Medicine, Faculty of Medicine, Chiang Mai University, Chiang Mai, 50200 Thailand; 6 Department of Judo Therapy, Faculty of Medical and Health Sciences, Tokyo Ariake University of Medical and Health Sciences, Tokyo, 135-0063 Japan; 7 Department of Health Service Support, Ministry of Public Health, Nonthaburi, 11000 Thailand; 8 Centre of Excellence for Health Economics, Faculty of Economics, Chulalongkorn University, Bangkok, 10330 Thailand

**Keywords:** Global health, Service delivery model, Population ageing, Health promotion, Asia, Universal coverage, Frailty, Implementation research

## Abstract

**Background:**

Populations around the world are ageing faster, with the majority living in low- and middle-income countries where health and social care are yet to be universal and inclusive for the ageing population. This community-integrated intermediary care (CIIC) model is a novel prevention-based, long-term care model enhancing the family-based care system traditionally practised in Thailand and neighbouring Asian countries, and many low-and middle-income countries globally. This study assessed the effectiveness of the CIIC model in Chiang Mai, Thailand.

**Methods:**

The two-arm parallel intervention study was designed as a cluster-randomized controlled trial. The study population at randomization and analysis was 2788 participants: 1509 in six intervention clusters and 1279 in six control clusters. The research protocol was approved by the WHO Research Ethics Review Committee (WHO/ERC ID; ERC.0003064).

The CIIC service intervention model is a combination of formal care and informal care in a subdistrict setting consisting of three components: (1) care prevention delivered as community group exercise and home exercise; (2) care capacity-building of the family caregiver; and (3) community respite service. The primary outcome was family caregivers’ burden at 6-month follow-up, and secondary outcome was activities of daily living. Analysis applied the intention-to-treat approach using cluster-level analysis via STATA 16 SE.

**Results:**

Baseline characteristics did not differ between the two arms. Loss of follow up was 3.7%. Mean age of the participants was 69.53 years. Women constituted 60%. The COVID-19 pandemic caused delayed implementation. The proportion of families with reduced caregiver burden at 6-month follow-up was higher among the intervention clusters (mean 39.4%) than control clusters (mean 28.62%). The intervention clusters experienced less functional decline and fewer people with depression.

**Conclusions:**

When communities are integrated for preventing care, and families are empowered for giving care, it is possible to secure universal access to health and social care for the older persons, with basic resources mobilized from communities. This study had shown the CIIC model as an effective and potential step to the realization of universal health and long-term care coverage being inclusive of ageing populations in Thailand and globally.

*Trial registration*: This trial was registered at the Thailand Clinical Trial Registry—Trial registration number TCTR20190412004, https://www.thaiclinicaltrials.org/#

## Background

Population ageing, in which over 60 years of age is defined as old age, is increasing in Thailand. It was 0.7 million (7.2%) in 2011, then rapidly increased to 11.5 million (almost 17%) in 2020 and is projected to be 33% by 2040 [1]. This rapid demographic change has brought a steep rise in the burden of age-related morbidities and disabilities due to noncommunicable diseases [2]. Recent reports have shown that an increasing number of older Thais have become home-bound and bed-bound [3]. The scope of long-term care comprises medical and nursing care, personal care services, assistance services and social services, enabling people to live either independently or in residential settings when they can no longer carry out routine activities of daily living on their own [4]. Although healthcare services are universally accessible and publicly financed for all Thai citizens, Thailand still has some way to go to in terms of long-term care policy due to the limited availability of formal care services and inequalities in access to those services. Such an imbalance poses the risk of pressure on hospital systems and excessive healthcare utilization as experienced in many countries [4]. Currently, long-term care in Thailand principally consists of informal care sectors which rely mainly on self-care and families in terms of providing care and funding, as well as working in partnership with communities and volunteers [5].

Families serve as the main caregivers for older Thai people. Filial responsibility is a social norm in Thai culture [6, 7]. There is intergenerational, two-way interdependency via financial remittance between adult children and grandparents raising grandchildren [8]. However, breadwinners and the middle-aged commonly migrate domestically for a better job and income, leaving their parents and the skipped generation families back at home in the rural area. As a result, nowadays there are few extended families still present in rural areas. With smaller family sizes and the increasing role of females in the labour market, the equilibrium of family care in Thailand is becoming a challenge to sustain. When families serve as the backbone of ageing care in fast-ageing countries, the steeply increasing caregivers’ burden negatively impacts on society through the loss of working hours, and even job loss, of caregivers [9, 10]. Policies supporting caregiving families are possible in terms of respite care, education and training, improving physical and mental well-being, family support programmes, financial support, and family care leave.

A recent analysis from the same study site showed that the time consumed with caregiving negatively impacted on the career of one in four family caregivers [11]. Furthermore, caregiver burnout can lead to abuse of the older person [12]. Interventions promoting the care capacity within the informal care sector may relieve the burden on these families. Additionally, vastly differing levels of education and care competencies found amongst family caregivers may cause inequalities in the care of older people across lower-resource and lower-income countries [13]. There are regular courses for caregivers in Thailand, but family caregiving remains a grey area in terms of meeting the needs of recipients. An ideal technical guidance package would address the different needs and issues of ageing persons. A recent study recruiting 867 older Thai adults  and their family caregivers revealed that 5.5% of family caregivers were overburdened and needing respite. The median income of the study participants was ฿9000 (Thai bahts). Respite service, in the form of CIIC, can be offered to family caregivers who become extremely burdened [14, 15]. A recent analysis showed that more than a quarter of family caregivers were willing to use respite services if they were accessible [14].

Given the inevitable increasing burden on family caregivers [6, 11, 16], it is important to strengthen the traditional family-based long-term care model by interventions that empower families, along with care prevention programmes to preserve the intrinsic capacity of the older persons. According to WHO, the concept of healthy ageing highlights the ability of older persons to do what they value doing, having intrinsic capacity and an age-friendly environment allowing for physical well-being, autonomy, social participation, and dignity. A recent survey in northern Thailand highlighted the profound need of care, with frailty (13.9%) and pre-frailty (50.9%) among the older persons [17]. The chance of frailty was higher amongst those with low-education and those living alone. This highlights the urgent need of care prevention in Thailand which has been overstretched for decades in terms of rehabilitation due to chronic diseases coupled with under-investment in active ageing.

Preventing frailty is possible through evidence-based community-integrated interventions [18]. Evidence in Japanese cohort studies showed that older people who participated in community-based group exercise programmes were less likely to be frail, delaying the onset of dependency [19]. Community group exercise not only promotes physical well-being and prevention of care need but also represents the path to active ageing favouring autonomy, social connection and quality of the life [20]. Such outcomes are important for the Thai older population as they are either self-reliant or family dependent. Thai communities are well-exposed to health promotion. The need is specifically to empower older community residents with a set of functional and suitable training exercises, comprising initial training of the techniques, time and space for community engagement and an affordable delivery model.

Universally accessible health and social care is an expectation of all second wave ageing countries like Thailand. In this study we created a novel ageing care model, that sought to enhance the family-based, long-term care system linked to primary healthcare services, integrated into the community and local government within each municipality. The CIIC model, serving as an intermediary centre, was situated and integrated within the community. It was hoped that the CIIC model would link families and communities to local formal services and funding. In addition, it was intended to promote active ageing through a community-based care prevention exercise programme for older persons. Moreover, the CIIC facility would serve as a small formal care home offering a short-term stay service by way of community-based respite care. The CIIC would deliver issue-specific, family caregivers’ training and assistance to enhance their caregiving capacity. It would target integration at different levels: (1) micro-level integration among the older persons, their families, their peer group and volunteers; (2) meso-level integration between the CIIC facility, the primary healthcare centre, community stakeholders and the municipality office; and (3) macro-level integration between the public health and public administration sectors under different ministries in Thailand (Table 1). Overall, the CIIC model sought to introduce an integrated package of formal long-term care services to the Thai community together with health promotion in order to boost family care capacity and prevent long-term care needs.

**Table 1:** Integration at different levels and resultant action or outcomes in the CIIC model

Level of integration	Integration	Action or outcome
Micro	Older persons Family caregivers Volunteer exercise trainer Health volunteers	1. Home exercise 2. Community-based, group care prevention functional exercise
Meso	CIIC facility Primary health care centre and professionals Community stakeholders Municipality office	1. Care capacity-building 2. Respite centre and formal care 3. Referrals to primary health care 4. Home visits for training family caregivers 5. Coordinating community-based activities 6. Data collection
Macro	Public health authority Public administration authority University academics WHO Japan International Cooperation Agency (JICA)	1. Building the CIIC facility 2. Establishment, implementation and sustainability of the model 3. Funding 4. Research

Whether the presence of a CIIC centre in a district can reduce the burden of family caregivers and promote the functional ability and quality of life (QOL) of older adults in the communities of Thailand is an interesting research question that has not yet been researched systematically. This study aimed to assess the effectiveness  of a CIIC model and compare it to the existing traditional family care model in Thailand. The objective of this study, therefore, was to assess the effectiveness of a CIIC service model that assisted families’ provision of long-term care for older adults in terms of the primary outcome, the family caregivers’ burden, and secondary outcomes, the older persons’ functional ability determined by their activities of daily living and QOL.

When it is compared to the existing model in Thailand or models in other low- and middle- or high-income countries, these factors constitute the uniqueness of the CIIC model.

- 1. CIIC is an integrated care model which consisted of (1) formal care offered to the community in the form of the community respite service and (2) informal care which is existing family care in the setting of the local community, and (3) prevention of long-term care needs through the care prevention exercise delivered in a population approach (Fig. 2).
- 2. The CIIC model applied screening of both older persons’ functional ability and family caregivers’ burden to grant the respite care and/or family caregiver support. In the setting where family-based care forms the main core of the long-term care for older people, it is necessary to measure the burden of family caregivers and launch intervention to reduce the burden of family caregivers. It is different from countries with an established long-term care system such as Japan where criteria for screening and granting the long-term care service do not incorporate screening the family caregiver burden.
- 3. There was an establishment of new facility for care coordination and service delivery in the CIIC model. The CIIC centre or facility is located within the community and serves as intermediary centre between the health centre which is providing healthcare services and the community.
- 4. The CIIC model collaborated with local government, as we thought future resource mobilization and sustainable finance will be contributed by the community and local government (Table 1).
- 5. A functional training exercise, which is a form of exercise to prevent the need for long-term care, to minimize functional decline, and a specific set for older persons, was introduced for the first time to the Thai community, although Thailand has existing aerobic group exercises for the general population. The exercise set in the CIIC model is particularly for older persons, including resistance training, stretching, balance training and evidence-based components, and designed for the Thai setting with stakeholders’ involvement. It targets enhancement of active ageing among the older independent community residents.
- 6. The CIIC centre refers medically diagnosed persons identified on screening, such as for hypertension and diabetes, to the primary healthcare centre. However, the CIIC model did not utilize the human resources of the primary healthcare centre for its function: neither human resources nor the budget. It paves the way for services insured in a different scheme such as universal health insurance and forthcoming long-term care insurance.

## Methods

### Design, setting and participants

The two-arm parallel intervention study was designed as a cluster-randomized controlled trial. It recruited 2788 participants: 1509 participants in six intervention clusters and 1279 participants in six control clusters. The research protocol was approved by the WHO Research Ethics Review Committee (WHO/ERC ID: ERC.0003064) dated 7 March 2019 and the Boromrajonani College of Nursing, Lampang Thailand Ethics Review Committee (E2562/005) dated 4 March 2019. It was registered at the Thailand Clinical Trial Registry under trial registration number TCTR20190412004 [21]. The trial was conducted over a period of 2 years. The net intervention period was 6 months.

### Study setting

The trial location was in Chiang Mai, Northern Thailand. A total of 284,457 older adults resided in Chiang Mai city according to a provincial report in 2019. This number accounts for 18% of its population and was higher than the national proportion of people aged 60 years and older, which was 16%. The culture in Chiang Mai is similar to that of many neighbouring Association of Southeast Asian Nations (ASEAN) countries in terms of caring for older parents as a family tradition and social value.

Two subdistricts were involved in the study: subdistrict XXX (10 villages) for the intervention arm, and subdistrict YYY (15 villages) for the control arm. They are in the same Mueang Chiang Mai district of Chiang Mai province. The distance between the two subdistricts was far enough to prevent relayed messages, although they were of a similar demographic and sociocultural characteristics. The two subdistricts have a similar population (more than 18,000) and similar ageing rates (more than 18% of people over 60). Residents in both subdistricts are mostly Thai people who have access to healthcare services under the national health insurance system and social welfare services for older people covered by municipality funds.

### Sample size and power

The estimated sample was 1500 participants in each arm to determine the effect size difference of 0.5 unit with standard deviation (SD) of 4 between the two arms. STATA version 16 SE (Stata Corporation, College Station, TX, USA) was utilized for sample and power estimations. The precision levels applied were a *P* value of 0.05 with a 95% confidence interval. The sample size was inflated for a design effect of 1.2 of cluster-randomized design application, and for compensation of potential nonresponses and drop-outs during the recruitment and study of up to 20%. Moreover, the sample size was estimated to be sufficient for detecting minimal difference in key parameters such as mean Caregiver Burden Inventory (CBI), activities of daily living (ADL) and health-related QOL indicators within the study population. We performed a backward sample power calculation at the end.

### Sequence generation, random allocation and blinding

**Fig. 1:**
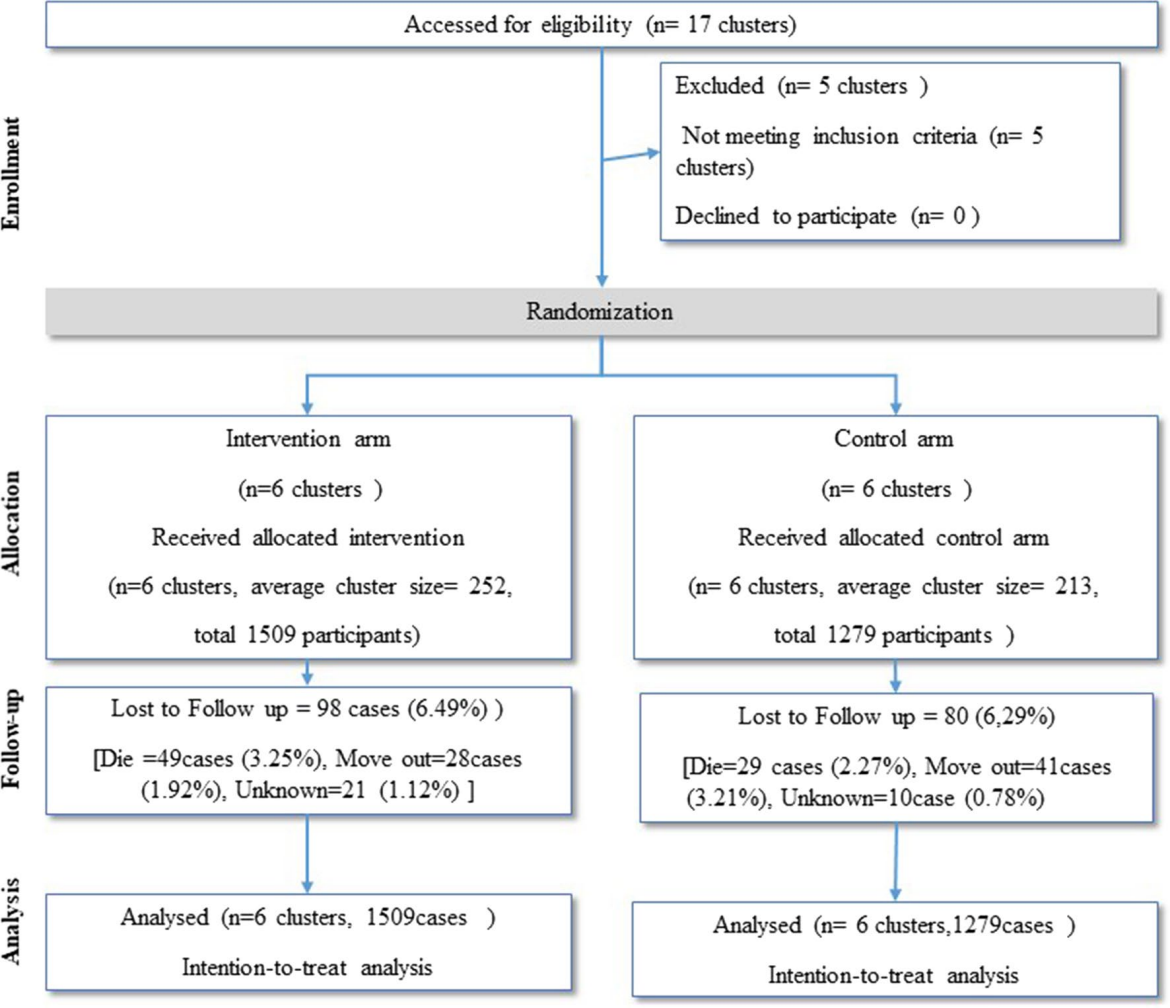
CONSORT (Consolidated Standards of Reporting Trials) diagram in the CIIC model for long-term care, Thailand

Cluster randomization was used to prevent contamination between the intervention subdistrict and control subdistrict. It was not possible to randomize the villages from different administrative areas and municipalities, and directly allocate them to the intervention and control arms. Therefore, we practised internal randomization to recruit six eligible clusters randomly within the intervention arm municipality, which has 10–15 villages, and likewise, in the control arm [22] (Fig. 1).

A statistician blinded to the study generated a random number for each arm of the study and recruited villages randomly within each arm. The control arm and intervention arm villages were geographically distant and administratively exclusive, yet they were in the same province and similar demographically, socially and culturally in order for social intervention evaluation.

It was not possible to do double blinding. However, participants were blind to allocation. Participants and research assistants carrying out assessment were not able to know the randomly allocated clusters before the study begun. Thus, bias and contamination were controlled.

The unit of randomization, cluster, was a village. Participants in the intervention clusters received the CIIC facility and service intervention after screening for eligibility. Eligibility criteria for a cluster was a village which had more than 300 older persons over 60 years of age at the time of randomization.

### Inclusion criteria for study participants

1. Persons over 60 years of age and their family caregivers
2. Either male or female
3. Resident in the study site districts.

### Exclusion criteria

1. Persons over 60 or their family caregivers without informed consent
2. Persons who could not understand the explanation for informed consent although provided with language support
3. Households without an older person over 60 years
4. People with cognitive impairment or severe impairment of decision-making abilities.

### Outcome measurement

Validated instruments commonly used in ageing and long-term care research were carefully chosen in order to assess the impact of the intervention in objective outcomes. Most of the instruments used were already translated into Thai versions and validated in previous studies and programmes in Thailand. We conducted a pilot test for the target population and ensured reliability of all the instruments in the study setting and context.

### Primary outcome

Primary outcome was the family caregiver’s burden at the 6-month follow-up. The CBI was used to measure burden of the caregiver [23, 24]. The CBI was applied to measure the family caregiver’s burden at baseline month 0 and month 6. The CBI is an internationally validated, 24-item, 5-point Likert scale which measures the caregiver burden in five dimensions as follows:

1. Time dependence burden (five items)
2. Developmental burden (five items)
3. Physical burden (four items)
4. Social burden (five items)
5. Emotional burden (four items).

After summing up the total score, a score greater than 36 indicates a risk of burnout, and a score ≥ 24 indicates a need to seek respite care.

### Secondary outcomes

Secondary outcomes consisted of biopsychosocial indicators such as functional ability, depression and QOL of the older persons, measured comparing the intervention and control arms after the first 6 months of intervention. Functional ability was assessed by applying Barthel’s ADL assessment [25]. ADL measures the level of ability for 10 basic items (bowels, bladder, grooming, toileting, feeding, transfer, mobility, dressing, stairs, bathing). Total possible scores range from 0 to 20, with lower scores indicating increased disability. Depression was screened by applying the Geriatric Depression Scale (GDS), which is commonly used internationally, validated and regularly used in Thailand [26]. Health-related QOL was measured through EuroQol 5-dimensions (EQ-5D) questionnaires [27]. A validated Thai version instrument was readily available and used after piloting. Health-related QOL (EuroQol 5-dimensions-5 levels [EQ-5D-5L]) with EQ visual analogue scale (EQ VAS) [27, 28] was applied to measure the health-related QOL of the older persons. The EQ-5D-5L comprises five dimensions: mobility, self-care, usual activities, pain/discomfort and anxiety/depression. Each dimension has five levels. The EQ VAS is a measure of overall self-rated health status. It records the respondent’s self-rated health on a vertical, visual analogue scale where the endpoints are labelled “Best imaginable health state” and “Worst imaginable health state”.

### Intervention

**Fig. 2:**
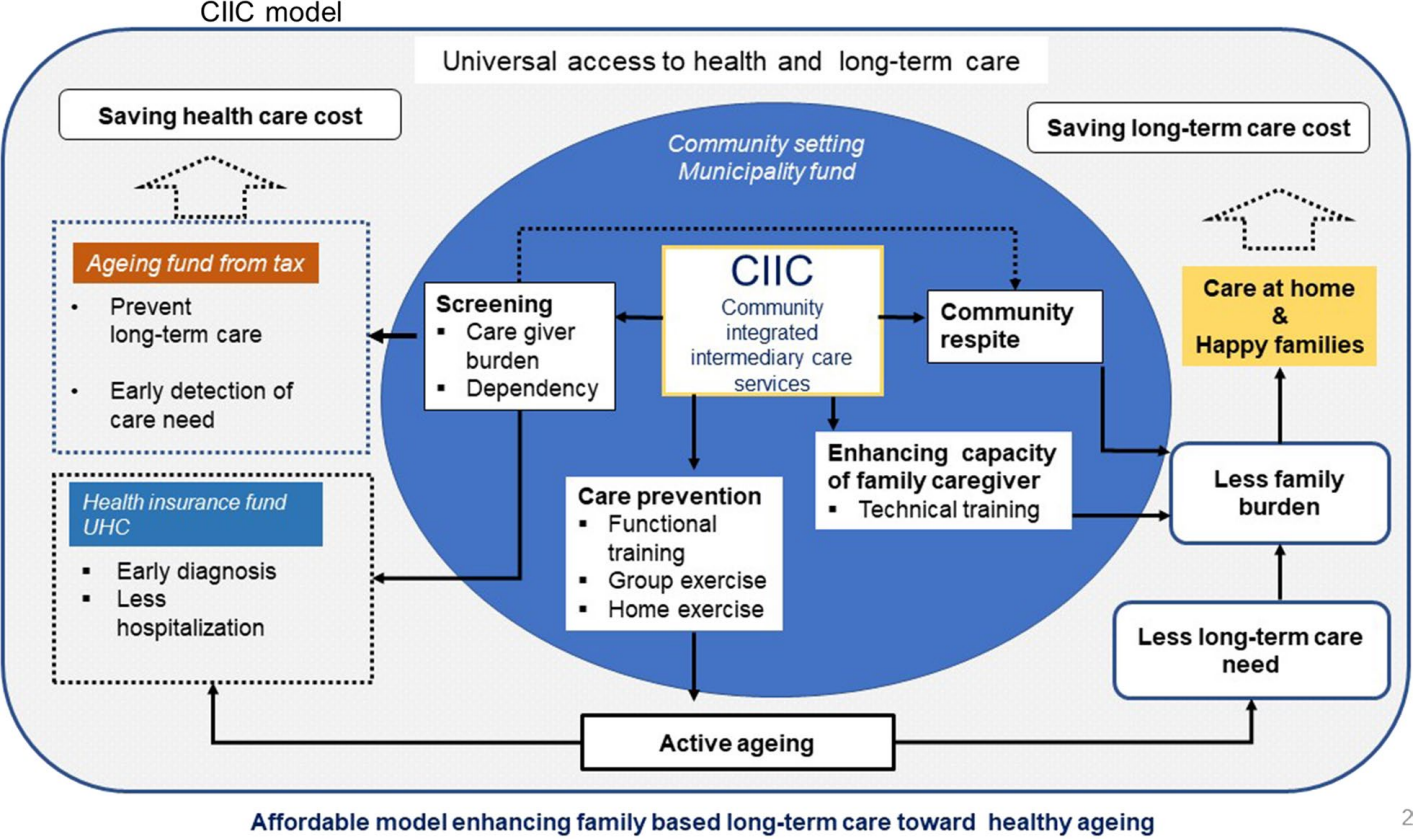
Service and contributions of the CIIC model explaining intervention components

The CIIC model is a novel prevention-based, long-term care model created to enhance the family-based long-term care system (Fig. 2) which has traditionally been practised in Thailand and its neighbouring Asian countries as well as many low- and middle-income countries globally [14, 29]. Conceptually, it is a combination of formal care and informal care in a district, subdistrict or a city. To implement the CIIC service model, a community-based facility had to be established within the community, mobilizing community resources and the municipality funds. It would serve as a 5–10-bed respite home and office for CIIC staffs. Two auxiliary nurses would work alternatively for care, coordination and capacity-building with two assistants and volunteers. The CIIC facility would be geographically community-centred, functionally linked to the primary healthcare centre and administratively linked to the municipality office. However, its human resources and professionals would be separate, not from the primary healthcare centre. The CIIC service model consisted of three components: (1) care prevention exercise, (2) care capacity-building of the family caregiver and (3) community respite service.

Care prevention exercise is to prevent the long-term care need of older persons, thus preserving their functional ability leading to active ageing (Fig. 2). It is a set of functional training exercises particularly designed for older adults either in a sitting or standing position. We provided two options: (a) 45-minute community-based group exercise and (b) 10-minute home exercise set for every day of the week to be practised twice a day individually or with friends and family at home. We provided an exercise DVD, wall posters to guide the exercise techniques, and a calendar for recording exercise. Home sets of exercises consisted of stretch, upper limb, lower limb, trunk and squat components [30], whereas group exercise consisted of dynamic and static stretch, tube training for upper and lower limbs, squat, balance training, walking and brain training. Community-based group exercise activity required a venue, schedule and a television or projector for a group of 30 persons. We created six such venues in the intervention clusters. Before launching the community-based group exercise, community volunteers were trained to be the leaders of group exercise. The training took 4 days. Stakeholders made the schedule of group exercises depending on the participant’s available time.

Secondly, the CIIC provided training and assistance to enhance the family caregiver’s care capacity (Fig. 2). The training was not a general, regular course. It was an issue-based technical training, relating to the need of the care recipient. CIIC teams arranged home visits when caring families made a request. A care expert nurse and the team reviewed the care techniques and taught the family caregiver on site. This activity was linked to the primary healthcare service, and community health volunteers coordinated with the municipality authority. A visit to home screened the care need of the older persons, the burden of the family caregiver and technical gaps, and the home modification need indicated what could be provided by the municipality and what could be referred to the primary healthcare centre.

The third service of CIIC was to provide formal long-term care service in the form of a community respite home for eligible older persons (Fig. 2). Eligibility criteria were set by the city stakeholders. In this study, the eligibility criteria for the CIIC temporary respite care service was for applicants with full-time, unpaid family caregivers caring for dependent individuals over 60 years of age and meeting the eligibility as assessed using the ADL and CBI measures [14].

### ADL criteria

In order to be eligible to be admitted to the CIIC facility, the family caregiver had to be personally providing the care recipient with assistance for at least two of the following six ADL: (1) bathing—the family caregiver is assisting the older adult with bathing, including help with washing, shampooing, getting in or out of the tub or shower, brushing teeth, and other aspects of personal grooming (bathing ADL score 0); (2) dressing—the family caregiver is assisting the older adult with dressing, including helping the individual put on or take off clothing and footwear (dressing ADL score 0 or 1); (3) toileting—the family caregiver is helping the older adult get on or off the toilet, commode or bedpan and clean themselves, or the individual is incontinent (toileting ADL score 0 or 1); (4) transferring—the family caregiver is helping the older adult get to and from a bed or chair (transfer ADL score 0 or 1); (5) walking or mobility—the family caregiver is helping the older adult move from one stationary point to another by removing obstacles, opening doors and assisting with canes, wheelchairs or other assistive devices (mobility ADL score 0 or 1); and (6) eating or feeding—the family caregiver is helping the older adult who has difficulty chewing or swallowing without assistance or needs partial or total help with eating (feeding ADL 0 or 1).

### CBI criteria

On the assessment of caregiver burden, the following two eligibility criteria were required to utilize the respite care service: (1) time dependency items score > 17 and (2) physical health items score > 14. If there was only one person in the family acting as a caregiver for the older adult with the qualifying ADL criteria and the caregiver had to leave the house to travel, the older adult would qualify for admission to the CIIC based on the available capacity of the facility on the appointment days. These criteria could be revised by stakeholders depending on the estimates of the population to be served, budget of the municipality, and capacity of the facility.

### Control group

The CIIC intervention was evaluated in comparison with an active comparator which was the existing service in Thailand. Control arm participants received the usual care (i.e. the current system of long-term care common to all provinces in Thailand), consisting principally of a health volunteers’ assistance to those living alone or severely dependent older persons in addition to traditional family-based care. The difference between the services of the two arms refers to the newly launched CIIC services, as described above.

### Screening and assessment of family burden and long-term care capacity

All older adults and caregivers in each cluster who provided informed consent were screened for ADL status and health-related QOL utilizing the EQ-5D-5L for older adults, and caregiver burden measured using the CBI. In both arms, a basic health check, blood pressure check and body mass index (BMI) assessment were provided by the study participants. If any disease was suspected through the health check, appropriate referrals to existing healthcare services were provided. This was a benefit for all study participants. The control group also received the same assessment with an explanation that the assessment was being conducted as part of a survey and the result would be utilized for research purposes. They also received the benefit of the additional health check followed by appropriate referrals to healthcare professionals.

### Data analysis

All analysis applied cluster-level analysis for all outcome measures. A cluster-adjusted *t*-test and cluster-adjusted chi-squared test were applied to compare the outcome between the intervention and control groups. At first, the result of the baseline survey was compared between the intervention and control arm clusters to check the balance between the two arms and to detect possible confounders (Table 2). We compared the level of the indicators measured at the baseline, applying cluster-level analysis. Similarity between the two arms after randomization indicated that cluster-adjusted analysis was sufficient.

**Table 2:** Baseline characteristic level of outcome indicator after randomization, by clusters in intervention and control arms

	Cluster	CBI		ADL		GDS		EQ index	
		Mean	SD	Mean	SD	Mean	SD	Mean	SD
Intervention	1	2.81	6.38	19.28	2.48	1.35	2.25	0.83	0.20
	2	14.36	13.16	19.43	1.97	2.11	2.32	0.72	0.23
	3	2.36	6.09	19.34	2.30	1.03	2.13	0.86	0.20
	4	1.48	4.67	19.23	2.30	1.14	1.99	0.89	0.16
	5	1.56	7.21	19.40	2.45	0.78	1.30	0.85	0.23
	6	6.63	9.83	18.76	3.17	2.76	2.69	0.75	0.28
	Mean	4.37	9.07	19.23	2.50	1.57	2.29	0.81	0.23
Control	7	4.43	8.61	18.99	3.21	1.44	2.31	0.78	0.26
	8	4.36	9.17	19.40	2.15	0.92	1.58	0.81	0.21
	9	4.69	9.94	19.33	2.34	0.85	1.46	0.84	0.16
	10	3.80	10.00	19.52	1.85	1.06	1.60	0.83	0.25
	11	3.90	9.31	19.06	2.76	0.97	1.61	0.78	0.24
	12	3.66	8.61	19.21	2.24	2.13	3.09	0.79	0.24
	Mean	4.15	9.46	19.25	2.46	1.23	2.07	0.81	0.23
ICC		0.052		0.017		0.019		0.047	
P		0.67		0.69		0.35		0.79	
MANOVA *P* value	0.08								

*CBI* Caregiver Burden Inventory scale, *ADL* activities of daily living score, *GDS* Geriatric Depression Scale, *EQ5D5L* European Quality of Life scale, *ICC* inter-cluster correlation, *MANOVA* multivariate analysis of variance, *P* *P* value of MANOVA test, *SD* standard deviation

Intention-to-treat analysis was applied. Biological, psychological and social indicators were compared between the two arms. The primary outcome was the CBI (Tables 3, 4, 5).

**Table 3:** Caregiver burden by clusters in intervention and control arms

	Clusters	Intervention arm						Control arm					
		Less burden CBI < 24		More burden 24–35		Most burden ≥ 36		Less burden CBI < 24		More burden 24–35		Most burden ≥ 36	
		*n*	%	*n*	%	*n*	%	*n*	%	*n*	%	*n*	%
	1	119	98.35	1	0.83	1	0.83						
	2	90	76.92	22	18.80	5	4.27						
	3	208	98.11	3	1.42	1	0.47						
	4	144	98.63	2	1.37	0	0.00						
	5	144	97.96	1	0.68	2	1.36						
	6	110	88.71	13	10.48	1	0.81						
	7							44	93.62	3	6.38	0	0.00
	8							143	96.62	2	1.35	3	2.03
	9							188	97.92	1	0.52	3	1.56
	10							178	95.70	2	1.08	6	3.23
	11							121	96.03	2	1.59	3	2.38
	12							103	96.26	1	0.93	3	2.80
Mean			93.11		5.6		1.29		96.03		1.98		2
ICC	0.0399 CI (0.0010, 0.0788)												
*P*	0.0001												

*CBI* Caregiver Burden Inventory scale, *ICC* inter-cluster correlation, *CI* 95% confidence interval, *P* *P* value of cluster-adjusted chi-squared test

**Table 4:** Proportion of families with reduced CBI, by clusters in intervention and control arms

	Clusters	Intervention arm				Control arm			
		CBI Not reduced		CBI Reduced		CBI Not reduced		CBI Reduced	
		*n*	%	*n*	%	*n*	%	*n*	%
	1	175	66.04	90	33.96				
	2	157	64.61	86	35.39				
	3	96	38.10	156	61.90				
	4	166	72.17	64	27.83				
	5	117	50.00	117	50.00				
	6	207	72.63	78	27.37				
	7					198	90.83	20	9.17
	8					114	55.88	90	44.12
	9					110	51.89	102	48.11
	10					145	63.32	84	36.68
	11					175	84.13	33	15.87
	12					171	82.21	37	17.79
Mean			60.6		39.4		71.38		28.62
ICC	0.0994 CI (0.02, 0.18)								
*P*	< 0.001								

*CBI* Caregiver Burden Inventory scale, *ICC* inter-cluster correlation, *CI* 95% confidence interval, *P* *P* value of cluster-adjusted chi-squared test

**Table 5:** CBI levels by clusters in intervention and control arms

	Clusters	Intervention arm		Control arm	
		Mean	SD	Mean	SD
	1	2.13	5.86		
	2	7.13	12.76		
	3	2.23	4.75		
	4	4.72	8.99		
	5	0.85	3.86		
	6	4.97	9.84		
	Mean	3.39	8.04		
	7			3.07	7.48
	8			1.07	2.57
	9			1.54	3.90
	10			4.03	9.54
	11			4.66	10.45
	12			3.72	7.31
	Mean			2.92	7.38
ICC	0.0527				
*P*	0.68				

*CBI* Caregiver Burden Inventory scale, *ICC* inter-cluster correlation, *P* *P* value of cluster adjusted chi-squared test, *SD* standard deviation

First, we compared outcomes in the categorical data. The CBI scores were categorized into three groups: a low burden (< 24), a medium burden (24–35), a high burden (≥ 36).

Second, we compared the change in CBI level between the baseline level and evaluation level. The decline in CBI within 6 months of follow-up was defined as a desirable outcome event (Table 4). In this analysis, those lost to follow-up or died were treated as a negative outcome in the intention-to-treat approach. Finally, we compared the level of the indicator, CBI measured at the evaluation, applying cluster-level analysis. The power of the sample was 0.79 for the primary outcome results. Applying a mixed-effect model, ICC for primary outcome was 0.08 (CI 0.03–0.18).

Effectiveness in preventing care need or functional decline was analysed using the secondary outcome ADL (Tables 6, 7, 8). First, it was analysed in three categories. Then the level of ADL was compared between intervention and control clusters. The change in ADL level was used to detect the outcome functional decline. Sustaining the same level of ADL or an increase in ADL level within 6 months of follow up was defined as a desirable outcome (Table 7). This analysis also applied an intention-to-treat approach and cluster-level analysis. Another secondary outcome, GDS, was analysed in categories and also comparing the level of GDS (Tables 9, 10). Health-related QOL was analysed comparing EQ5D5L level between the intervention and control arms in cluster-level analysis (Table 11).

**Table 6:** ADL among three groups in the intervention and control clusters

	Clusters	Intervention arm						Control arm					
		ADL ≥ 12		5–11		0–4		ADL ≥ 12		5–11		0–4	
		*n*	%	*n*	%	*n*	%	*n*	%	*n*	%	*n*	%
	1	229	97.03	3	1.27	4	1.69						
	2	220	96.07	4	1.75	5	2.18						
	3	237	95.95	9	3.64	1	0.40						
	4	215	99.08	0	0.00	2	0.92						
	5	218	97.32	4	1.79	2	0.89						
	6	242	93.80	8	3.10	8	3.10						
	7							202	95.28	4	1.89	6	2.83
	8							169	96.02	5	2.84	2	1.14
	9							191	95.98	7	3.52	1	0.50
	10							213	96.82	6	2.73	1	0.45
	11							183	93.85	11	5.64	1	0.51
	12							182	92.39	15	7.61	0	0.00
Mean			96.54		1.93		1.53		95.06		4.04		0.9
ICC	0.0014 CI (0.0000, 0.0064)												
*P*	0.0035												

*ADL* activities of daily living score, *ICC* inter-cluster correlation, *CI* 95% confidence interval, *P* *P* value of cluster-adjusted chi-squared test

**Table 7:** Proportion without ADL decline among the intervention and control clusters

	Clusters	Intervention arm				Control arm			
		ADL declined		Care prevented		ADL declined		Care prevented	
		*n*	%	*n*	%	*n*	%	*n*	%
	1	46	17.36	219	82.64				
	2	42	17.28	201	82.72				
	3	49	19.44	203	80.56				
	4	23	10.00	207	90.00				
	5	33	14.10	201	85.90				
	6	67	23.51	218	76.49				
	7					53	24.31	165	75.69
	8					40	19.61	164	80.39
	9					30	14.15	182	85.85
	10					47	20.52	182	79.48
	11					53	25.48	155	74.52
	12					52	25.00	156	75.00
Mean			16.95		83.05		21.51		78.49
ICC	0.0107, CI (0.0000, 0.0232)								
*P*	0.0043								

*ADL* activities of daily living score, *ICC* inter-cluster correlation, *CI* 95% confidence interval, *P* *P* value of cluster-adjusted chi-squared test

**Table 8:** ADL levels by clusters in intervention and control arms

	Clusters	Intervention arm		Control arm	
		Mean	SD	Mean	SD
	1	17.06	6.49		
	2	18.00	5.31		
	3	18.58	3.74		
	4	18.41	4.96		
	5	18.56	4.50		
	6	16.78	6.44		
	Mean	17.85	5.43		
	7			18.19	4.33
	8			16.27	6.97
	9			18.07	5.08
	10			18.33	4.31
	11			17.52	5.20
	12			17.48	4.97
	Mean			17.67	5.23
ICC	0.0173				
*P*	0.69

**Table 9:** State of depression GDS three groups in two clusters

	Clusters	Intervention arm						Control arm					
		Normal 0–4		Mild depression 5–10		Severe depression 11–15		Normal 0–4		Mild depression 5–10		Severe depression 11–15	
		*n*	%	*n*	%	*n*	%	*n*	%	*n*	%	*n*	%
	1	221	93.64	15	6.36	0	0.00						
	2	196	86.34	31	13.66	0	0.00						
	3	224	90.69	23	9.31	0	0.00						
	4	186	86.11	29	13.43	1	0.46						
	5	203	90.63	21	9.38	0	0.00						
	6	197	76.36	59	22.87	2	0.78						
	7							180	84.91	24	11.32	8	3.77
	8							159	90.34	15	8.52	2	1.14
	9							171	86.36	16	8.08	11	5.56
	10							195	88.64	17	7.73	8	3.64
	11							171	87.69	15	7.69	9	4.62
	12							153	77.66	29	14.72	15	7.61
Mean			87.3		12.5		0.21		85.93		9.68		4.39
ICC	0.0218 (0.0002, 0.0434)												
*P*	< 0.0001												

*GDS* Geriatric Depression Scale, *ICC* inter-cluster correlation, *CI* 95% confidence interval, *P* *P* value of cluster-adjusted chi-squared test

**Table 10:** The level of GDS by clusters in intervention and control arms

	Clusters	Intervention arm		Control arm	
		Mean	SD	Mean	SD
	1	3.28	4.41		
	2	3.13	3.55		
	3	2.62	2.39		
	4	3.14	3.50		
	5	2.52	3.07		
	6	4.46	4.08		
	Mean	3.23	3.64		
	7			2.34	3.79
	8			3.50	5.20
	9			3.05	4.61
	10			2.33	3.91
	11			2.78	4.54
	12			3.33	4.90
	Mean			2.87	4.52
ICC	0.026				
*P*	0.23				

*GDS* Geriatric Depression Scale, *ICC* inter-cluster correlation, *P* *P* value of cluster-adjusted chi-squared test, *SD* standard deviation

**Table 11:** EQ5D5L by clusters in intervention and control arms

	Clusters	Intervention arm		Control arm	
		Mean	SD	Mean	SD
	1	0.79	0.29		
	2	0.82	0.23		
	3	0.84	0.19		
	4	0.81	0.27		
	5	0.77	0.28		
	6	0.75	0.28		
	Mean	0.80	0.026		
	7			0.79	0.26
	8			0.78	0.28
	9			0.82	0.25
	10			0.83	0.22
	11			0.87	0.22
	12			0.75	0.31
Mean				0.80	0.26
ICC	0.0190				
*P*	0.76				

EQ5D5L: European Quality of Life scale, ICC: inter-cluster correlation, CI: 95% confidence interval: *P*: *P* value of cluster-adjusted chi-square test, SD: standard variation

## Results

The number of participants at randomization was 1509 in the intervention arm and 1279 in the control arm. The number of participants at the analysis was 1460 (95.92%) in the intervention arm and 1250 (96.65%) in the control arm. A total of 49 (3.25%) people died in the intervention arm, and 29 in the control arm (2.27%). The overall loss of follow-up was 100 (3.7%) people because of movement to other regions and loss of contact.

The mean age of the study participants was 69.53 years, with women representing 60% of the participants. A total of 10.3% of the participants were living alone, while 33.9% were staying together with their spouse, 42.9% with children and 5.3% with siblings. Sixty-two percent of the participants were primary school educated, 31% achieved secondary and tertiary level education, while only 7% had no formal education. Thirty percent of the participants were still working in an employed job.

### Baseline characteristics

When comparing baseline levels of CBI, ADL and GDS between the intervention and control arms, there were no significant differences (*P* = 0.08 MANOVA). Randomization was successful. We analysed all three outcomes in the MANOVA model, and they were not statistically significant (Table 2).

### Primary outcome: effectiveness in reducing the CBI

Effectiveness of the intervention was assessed applying the CBI as the primary outcome of the study.

1. The proportion of high-burdened families (CBI score ≥ 36) was higher in the control arm clusters than the intervention arm clusters. It was statistically significant (*P* < 0.001, ICC 0.03, cluster-adjusted chi-squared test) (Table 3).
2. The change in the CBI was assessed according to the proportion of families whose CBI was reduced by the intervention (Table 4). The proportion of families with a reduced caregiver burden at the 6-month follow-up was higher among the intervention clusters (mean 39.4%) than the control clusters (mean 28.62%). It was statistically significant (*P* < 0.001, ICC 0.01, cluster-adjusted chi-squared test).
3. We compared the level of CBI between the intervention and control arms at the evaluation, at 6 months after the intervention. The difference in the levels of CBI between the intervention and the control clusters was statistically not significant (*P* = 0.68, ICC 0.05 cluster-adjusted *t*-test) (Table 5). It was also not significant statistically in the generalized estimating equation (GEE) model.

Mean CBI scores, particularly social burden and emotional burden, were significantly reduced by the intervention of CIIC. Average CBI scores of “social burden” at pre- and post-intervention were (0.71 ± 1.94) vs (0.45 ± 1.50), (*P* 0.029), and those of “emotional burden” at pre- and post-intervention were (0.79 ± 2.01) vs (0.60 ± 1.86), (*P* 0.001).

### Secondary outcomes

#### Effectiveness in preventing functional decline

1. The proportion of ADL decline was higher in the control arm clusters than in the intervention arm clusters. It was statistically significant (*P* = 0.004, ICC 0.001, cluster-adjusted chi-squared test) (Table 6).
2. Change in ADL: The proportion of participants without functional decline at the 6-month follow-up was higher among the intervention clusters (mean 83%) than the control cluster (mean 78%). The preventive effect was statistically significant (*P* = 0.004, ICC 0.001, cluster-adjusted chi-squared test) (Table 7).
3. The difference in the levels of ADL between the intervention and the control clusters was statistically not significant (*P* = 0.069, ICC 0.017, cluster-adjusted *t*-test) (Table 8). It was also not significant statistically in the GEE model.

Mean ADL scores before and after intervention of CIIC were significantly different among nine domains such as “Grooming” (0.99 ± 0.12) vs (0.97 ± 0.16) (*P* 0.01), “Bathing” (0.98 ± 0.14) vs (0.97 ± 0.17) (*P* 0.02), “Dressing” (1.96 ± 0.24) vs (1.94 ± 0.30) (*P* < 0.001), “ Mobility” (2.92 ± 0.38) vs (2.89 ± 0.47) (*P* 0.003), “ Stairs” (1.91 ± 0.35) vs (1.88 ± 0.43) (*P* < 0.001), “Transfer” (2.93 ± 0.36) vs (2.88 ± 0.45) (*P* < 0.001), “Toilet use” (1.96 ± 0.27) vs (1.92 ± 0.34) (*P* < 0.001), “Bowel control” (1.91 ± 0.36) vs (1.87 ± 0.38) (*P* 0.003) and “Bladder control” (1.85 ± 0.42) vs (1.81 ± 0.43) (*P* 0.001), respectively.

#### Effectiveness in preventing depression

1. The proportion of participants without depression was higher among the intervention clusters (mean 87.14%) than the control clusters (85.89%). It was statistically significant (*P* < 0.001, ICC 0.02, cluster-adjusted chi-squared test) (Table 9).
2. The level of GDS was not significantly different between the intervention and control clusters (*P* = 0.23, ICC 0.03, cluster-adjusted *t*-test) (Table 10).

#### QOL

QOL measured according to the EQ5D5L level between the intervention and control arm clusters was not significantly different (*P* = 0.076, ICC 0.02, cluster-adjusted *t*-test) (Table 11).

#### Intervention Cost of CIIC

**Fig. 3:**
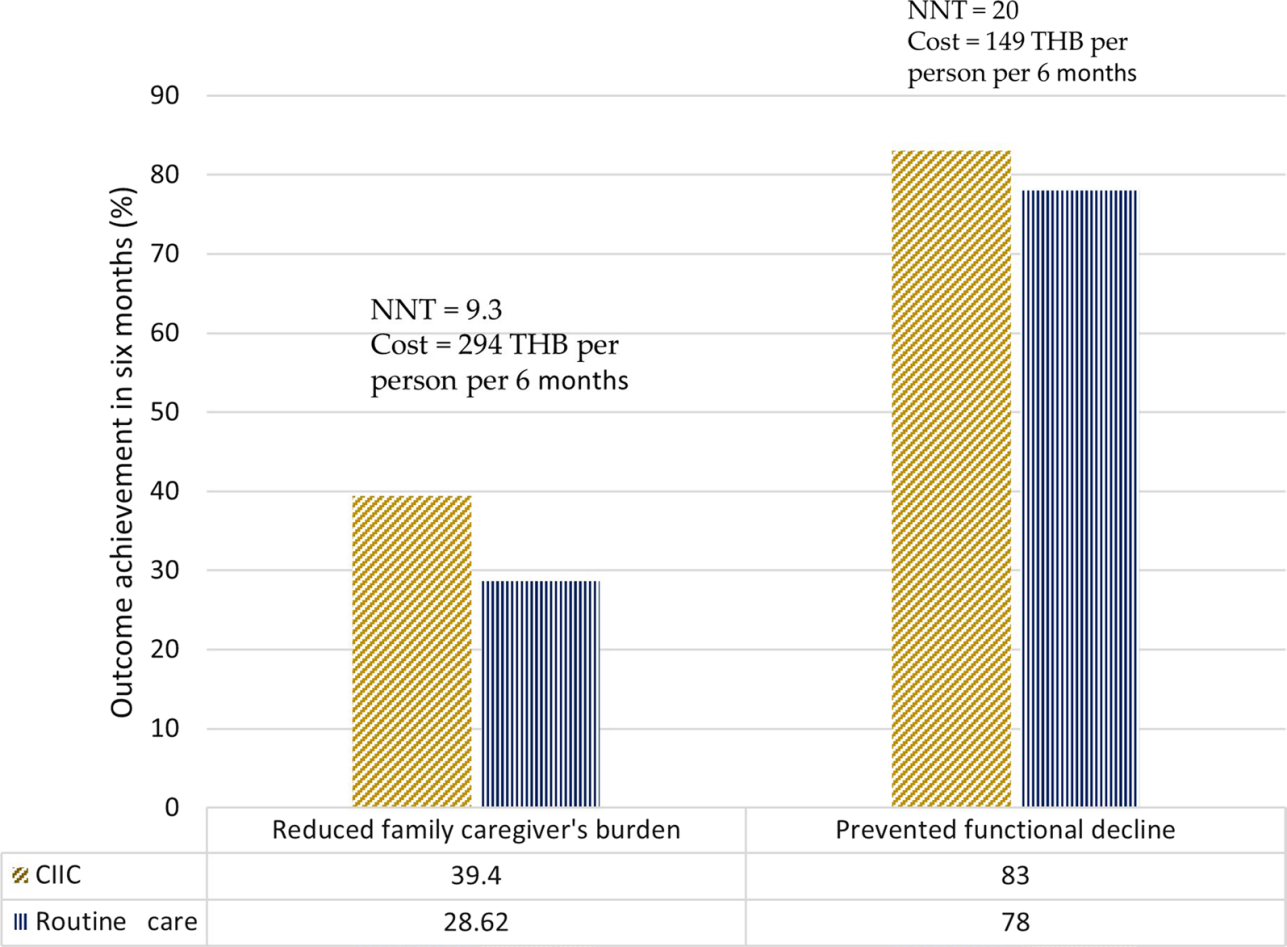
Effectiveness in 6 months and cost of enhancing family care capacity and long-term care prevention intervention components in the CIIC trial during the COVID-19 pandemic, Thailand 2021 (*n* = 2788), THB Thai baht (tentatively 1 THB = 0.03 USD as of current exchange rate)

The number needed to treat (NNT) for reducing the family burden was 9.3 (Table 4; Fig. 3), and the NNT for care prevention was 20 (Table 7; Fig. 3). We calculated the cost of each component of CIIC intervention in terms of capital cost, operational labour cost and operational material cost. The cost to provide care prevention intervention is 149 THB per 6 months per person. In relation to reducing the family burden, the cost of providing family caregiver capacity-building and standby respite care is 669 THB per 6 months per person and 294 THB per 6 months per person without respite care (Fig. 3).

## Discussion

Populations around the world are ageing faster. In 2021, at the start of global healthy ageing decade, there were more than 1 billion people aged over 60 years around the world, with the majority living in low- and middle-income countries, where health and social care are yet to be fully inclusive of the ageing population [31]. Furthermore, multiple barriers confront the full participation of these older people in society. Community-integrated innovative models, which would secure access to health and social care for the older persons, providing the basic resources necessary for a life of meaning and dignity, are urgently required. The results of this cluster-randomized controlled trial, carried out in the setting of Chiang Mai, Northern Thailand, have shown that CIIC is an intervention model which may be effective in preventing long-term care need (Table 7) and in reducing the burden on family caregivers (Table 4) amidst the ongoing COVID-19 pandemic. It can enhance the capacity of the caring families, which constitutes the core of the existing traditional long-term care model (Fig. 3). Moreover, it may serve to prevent the long-term care need through community-integrated health promotion by preserving the functional capacity of older persons (Fig. 3).

It is worth discussing the details of primary outcome. We published the baseline data analysis that older age of family caregivers and diabetic family caregivers are associated with a high level of caregiver burden [11]. Family caregiver burden of cluster 2 of the intervention arm was the highest among other clusters because of caregivers’ older age and higher diabetes mellitus prevalence. About 62.4% of family caregivers in this cluster were older than 60 years of age which was the highest percentage of older caregivers when compared to other clusters with 29.7% being the lowest. The association between the caregiver’s physical health and stressors as they aged could explain the increased burden among older family caregivers. We noticed that cluster 2 in the intervention arm included 33.3% of family caregivers having diabetes, the highest among all clusters, whereas the lowest proportion of diabetic caregivers in other clusters was about 2.4%. Association of diabetes with higher caregiver burden was reported in the baseline data analysis and prior publication. It might be due to the added burden of managing their own diabetes along with taking care of their older care recipients [11]. Despite such hurdle, intervention in CIIC relieved the burden of family caregivers (Fig. 2; Tables 3, 4).

Health and social care in the community is the dream for many developing countries around the world. The increasing role of municipalities to be involved in health and long-term care, within a decentralized approach, requires a community-integrated model which is effective and sustainable in terms of the municipality setting and funding [32]. CIIC is a new concept and a new service model based on preventive strategy implemented through community integration (Fig. 2). The type of service is intermediary. It is a combination of formal care and informal care in a particular community, subdistrict, district, city or country. It is an integration of primary healthcare, social care and health promotion in the community setting (Fig. 2). Locating CIIC services in a neighbourhood environment is designed to raise local attention and people’s awareness, familiarity and responsibility within the social network of the local community.

The results of this study saw a significant reduction of the caregiver burden in the intervention arm (Table 4). CIIC provided technical guidance and advice for caregiving depending on the needs of the families with dependent older adults. CIIC nurses and volunteers visited participants’ houses and trained them in caregiving techniques. CIIC services to enhance the family caregiver’s capacity were provided to home-bound patients in integration with the primary healthcare centre. Caregivers could acquire formal information through the CIIC team, especially from the nurse and training of the tasks which require manual dexterity and technique to carry them out, such as hygiene tasks (bed bathing), transfer and mobility. As a result, the family caregiver’s burden decreased in the intervention arm in comparison with the routine care in Thailand. Improving the caregiver’s skills, together with providing mental support via access to short-term respite care services in the local community when feeling burdened or burnout could relieve the burden on those informal caregivers. There was less respite home admission, whereas home visits and community outreach became the main service. The determining factor was the safety concern for facility-based care during the COVID-19 pandemic. Evidence in other settings also reported the increasing utilization of home-based and family-based services during the COVID-19 pandemic [33].

Moreover, caregiver burden measurement of the family caregivers can be applied as a screening tool to assess long-term care needs, complementing the routine dependency assessment of care recipients using ADL scoring [11, 14]. In many developing countries, studies increasingly have reported the long-term care need among older persons [34, 35]. National programmes have started to screen for frailty among the older persons with several kinds of frailty screening tools. In a setting where long-term care is mainly provided through unpaid, informal care by families, the decision to grant formal long-term care service will require assessment of the family caregiver burden, in addition to the older person’s care need [14, 36]. Moreover, the assessment should be applicable in the community setting as understandable evidence for stakeholders.

In this intervention study, we introduced three steps as part of a prevention strategy employing CIIC services. The first step was a co-creation of community group exercise for prevention of long-term care need. Participation in such community exercises kept the seniors active, connected and independent, with the potential outcome that long-term care need will be reduced [22]. Home exercise, which was designed as an alternative to group exercise, became a major breakthrough as an intervention to sustain care prevention during the COVID-19 pandemic.

Functional training exercises have been reported as effective ways to prevent frailty [37]. Effectiveness in this study related to the delivery model that we used to introduce care prevention exercise to the Thai community. Community volunteers were trained to be the leaders for group exercise activities, with training lasting 4 days. The schedule, place and time were determined according to the community stakeholder’s meeting and older persons’ decision. The researcher introduced the techniques and required devices, the protocol for safety, and the alternative home exercise option. Municipality officers coordinated these events. Recently, the WHO Centre for Health Development referred to such activities as community-based social innovation (CBSI) for healthy ageing [38]. CBSI in Thailand is usually part of a state-driven model. In the CIIC model, the technique and devices were offered from the research, but community members decided everything else. Practically, it promoted autonomy, civic participation, and dignity of the older persons [39]. CIIC applied community empowerment for launching care prevention exercise to prevent frailty. Consequently, the burden on the caregivers decreased (Tables 3, 4, 5, 6, 7).

Second, when there is little care need, the older people can enjoy staying at home with their families, thereby improving their chances of “ageing in place” [4]. Families were assisted through training, direct help and respite service provided by the CIIC [9]. When family caregivers are faced with extreme burden and burnout, the older persons will be able to access a formal care service in the community. Again, the hope is that it can prevent unnecessary long-term care costs. CIIC offers an affordable model integrating formal services and informal care by families (Fig. 3). It may be applicable in many resource-limited countries helping to establish a fully structured, formal long-term care service.

Third, when older people remain active and healthy, their healthcare needs may be reduced, thereby avoiding unnecessary hospital admission and reducing healthcare costs. Further analysis of CIIC for cost and benefits will be required. Despite the higher stress due to the COVID-19 pandemic, older residents who had CIIC in their communities were less likely to get frail, compared to the residents in the control sites. Prevention of care need also reduced the burden on family caregivers (Tables 4, 7). Therefore, this CIIC model may promote active ageing in place and prevent the long-term care need and healthcare costs.

In addition, the screening of activities of daily living and of common noncommunicable diseases was able to identify unmet needs earlier and so prevent age-related morbidity and disability. Frailty screening is haphazardly carried out by primary healthcare centres. CIIC introduced a dedicated facility and staff for screening frailty, which is internationally recommended [40, 41]. Therefore, in the long term, the interaction of CIIC services and primary healthcare services could be harmonized, leading to better integration and representing a step to realizing universal access to long-term healthcare in Thailand.

### Lesson in implementation

A range of lessons were learned in developing and implementing CIIC. Researchers sought shared value with partners such as the municipality authority, primary healthcare providers, and the Ministry of Public Health. Initial advocacy applied a shared narrative to explain why integrated care matters. A persuasive vision was described to stakeholders about what CIIC could achieve. It is because we established shared leadership among the study site municipality administration, the public health authority and the researchers that communities could find the venue, time, professionals and volunteers and mobilize resources to develop a community respite facility and coordination of community-based care prevention activities for older persons. This represented a new understanding and a new way of working. The services of CIIC, such as care prevention, empowerment of family caregivers, and community respite, were well-identified, and the older community residents could see the real benefits from the integrated services.

The study is not without limitation. We reported the effectiveness of the model, but the nature of multiple intervention components in the model limited us to describing the effect of each intervention component one by one.

Moreover, community-integrated care was developed both from the bottom up, mobilizing community resources, and from the top down, triggering interest, commitment and involvement of the public health authority. CIIC could support and empower caregivers and older people to take more control over their health and well-being. Formal and informal sectors were integrated effectively in an innovative model to maintain the intrinsic capacity of older persons and to reduce family caregiver burden, leading to a specific objective of a healthy ageing community, with realistic costs of integrated care. If the model can be applied and sustained in the community, routine identification of frailty and risk stratification can be readily applied.

However, the COVID-19 pandemic delayed the intervention and reduced the participation. After 6 months of CIIC services, there was a significantly lower caregiver burden. The COVID-19 pandemic impeded the implementation and might have reduced the effect size (Tables 5, 8, 11). Despite such a situation and comparing to active control, CIIC interventions saw effectiveness in enhancing family care capacity and preventing long-term care, with low cost in a 6-month implementation period.

Facing the increasing burden of noncommunicable diseases and age-related morbidity, Thai communities are increasingly in need of community-integrated care models for older persons that can link existing health systems and reduce the burden upon caring families. This need is common to many countries in the ASEAN and around the world. The role of informal caregiving is more emphasized in high-income countries recently, whereas there is very little formal caregiving capacity in low-income countries. Since ASEAN and many Asian countries share similar traditional family-based, long-term care systems, the protocol, implementation experiences, results and products from CIIC may provide evidence-based policy options for other countries wishing to adopt similar community-integrated care models for older people.

The trial was carefully designed to have enough power to see intervention effectiveness compared to the active control. This CIIC trial implementation was impacted by the COVID-19 pandemic, as with many trials across the world. However, we retained a large sample at randomization and analysis. Our backward calculation revealed that the power of the sample at analysis was 0.79 which was adequate for the effect size of the finding relating to the primary outcome of the caregiver burden. Within a short implementation time of 6 months, interrupted by the pandemic-related situations, we could see the effectiveness of the community-integrated intermediary model in reducing the caregivers’ burden (Table 4) and preventing functional decline of older people (Table 7). Yet, the effectiveness of CIIC as reported in this report may only represent the tip of the iceberg. Sustainability and maintenance of implementation is required to see the full effectiveness and provide stronger evidence for the ageing world. This intervention study of a comprehensive model, well-integrated into the community, is the first to have been carried out in Thailand and represents an initiative for the rest of Asia and the world. Opportunities for scaling up CIIC in future research remain.

## Conclusion

This study showed the CIIC model to be an effective and potential step to the realization of universal health and long-term care coverage being inclusive of the ageing population in Thailand. The evidence and lessons learned in this study are expected to be beneficial in order to scale up CIIC across Thailand, other similar countries in Asia and around the world.

## Data Availability

The datasets used and analysed during the current study are available from the corresponding author on reasonable request.

## Abbreviations

ADL: Activities of daily living
ASEAN: Association of Southeast Asian Nations
CBI: Caregiver Burden Inventory
CIIC: Community-integrated intermediary care
GDS: Geriatric Depression Scale
MANOVA: Multivariate analysis of variance
QOL: Quality of life
SD: Standard deviation
